# Modifying Saccharomyces cerevisiae Adhesion Properties Regulates Yeast Ecosystem Dynamics

**DOI:** 10.1128/mSphere.00383-18

**Published:** 2018-10-24

**Authors:** Debra Rossouw, Skye P. Meiring, Florian F. Bauer

**Affiliations:** a Institute for Wine Biotechnology, Stellenbosch, South Africa; b Department of Viticulture and Oenology, Stellenbosch University, Matieland, South Africa; Carnegie Mellon University

**Keywords:** adhesion, cell-cell interaction, interspecies, yeast

## Abstract

Physical contact between yeast species, in addition to better-understood and reported metabolic interactions, has recently been proposed to significantly impact the relative fitness of these species in cocultures. Such data have been generated by using membrane bioreactors, which physically separate two yeast species. However, doubts persist about the degree that the various membrane systems allow for continuous and complete metabolic contact, including the exchange of proteins. Here, we provide independent evidence for the importance of physical contact by using a genetic system to modify the degree of physical contact and, therefore, the degree of asexual intraspecies and interspecies adhesion in yeast. Such adhesion is controlled by a family of structurally related cell wall proteins encoded by the *FLO* gene family. As previously shown, the expression of specific members of the *FLO* gene family in Saccharomyces cerevisiae dramatically changes the coadhesion patterns between this yeast and other yeast species. Here, we use this differential aggregation mediated by *FLO* genes as a model to assess the impact of physical contact between different yeast species on the relative fitness of these species in simplified ecosystems. The identity of the *FLO* gene has a marked effect on the persistence of specific non-Saccharomyces yeasts over the course of extended growth periods in batch cultures. Remarkably, *FLO1* and *FLO5* expression often result in opposite outcomes. The data provide clear evidence for the role of physical contact in multispecies yeast ecosystems and suggest that *FLO* gene expression may be a major factor in such interactions.

**IMPORTANCE** The impact of direct (physical) versus indirect (metabolic) interactions between different yeast species has attracted significant research interest in recent years. This is due to the growing interest in the use of multispecies consortia in bioprocesses of industrial relevance and the relevance of interspecies interactions in establishing stable synthetic ecosystems. Compartment bioreactors have traditionally been used in this regard but suffer from numerous limitations. Here, we provide independent evidence for the importance of physical contact by using a genetic system, based on the *FLO* gene family, to modify the degree of physical contact and, therefore, the degree of asexual intraspecies and interspecies adhesion in yeast. Our results show that interspecies contact significantly impacts population dynamics and the survival of individual species. Remarkably, different members of the *FLO* gene family often lead to very different population outcomes, further suggesting that *FLO* gene expression may be a major factor in such interactions.

## Abstract (precis)

The impact of direct (physical) versus indirect (metabolic) interactions between different yeast species has attracted significant research interest in recent years. This is due to the growing interest in the use of multispecies consortia in bioprocesses of industrial relevance and the relevance of interspecies interactions in establishing stable synthetic ecosystems. Compartment bioreactors have traditionally been used in this regard but suffer from numerous limitations. Here, we provide independent evidence for the importance of physical contact by using a genetic system, based on the *FLO* gene family, to modify the degree of physical contact and, therefore, the degree of asexual intraspecies and interspecies adhesion in yeast. Our results show that interspecies contact significantly impacts population dynamics and the survival of individual species. Remarkably, different members of the *FLO* gene family often lead to very different population outcomes, further suggesting that *FLO* gene expression may be a major factor in such interactions.

## INTRODUCTION

Microbial cell walls are the cells’ primary interface with the environment and other organisms. Research into the interactions between different yeast species indicates that direct physical contact between cells contributes significantly to ecological interactions, such as inhibition or stimulation (1, 2). It has been suggested that the early death of two non-Saccharomyces yeasts, namely Kluyveromyces thermotolerans and Torulaspora delbrueckii, in mixed fermentations with S. cerevisiae was due to cell-to-cell contact (1). Lopez et al. (3) similarly assessed the direct and indirect interactions between two yeast species, namely S. cerevisiae and Kluyveromyces marxianus, and found that both were inhibited in terms of growth and cell numbers only when in direct contact with one another. However, the systems used to establish the importance of physical contact in interspecies interactions in yeast have been based on the use of so-called membrane bioreactors. In these systems, two species are inoculated in separate compartments separated by a membrane, designed in such ways as to allow for the exchange of metabolites but not for mixing of cells. Other systems use different membranes and manners to ensure metabolic homogeneity between the compartments, including using peristaltic pumps (2) and other tools. These systems do not allow for an immediate and complete transfer of all relevant metabolites and macromolecules, such as proteins, which poses limitations on the nature and extent of the interactions investigated. Furthermore, no data exist regarding the mechanisms or genes that are involved in supporting physical interaction-driven fitness. Investigating genes or gene families which could potentially modulate or regulate interspecies cell-cell contact would be ideal to fill this knowledge gap. This would allow for controlled, directed physical interactions between selected species, enabling the determination of viability impacts on the species involved, compared with noninteracting control scenarios.

*FLO* genes, which encode cell wall-anchored adhesion proteins are mostly, if not entirely, responsible for the modifications of sex-independent adhesion properties of yeast cell walls (4, 5). Flo proteins are lectin-like proteins which bind to cell wall mannans on adjacent cells (6–8). In this process, Ca^2+^ ions act as cofactors in maintaining the active conformation of surface proteins, thereby enhancing the capacity of lectins to interact with α-mannan carbohydrates (9).

In S. cerevisiae, *FLO* genes are represented by a family of subtelomeric genes (*FLO1*, *FLO5*, *FLO9*, and *FLO10*) as well as the nonsubtelomeric gene *FLO11*/*MUC1*. The different Flo proteins are structurally very similar, and all data thus far show a strong functional overlap in terms of broad phenotypic impacts (4, 10, 11). Recently, it has been suggested that the *FLO* gene family may be involved in building niche ecosystems or associations of different yeast species in natural ecosystems (12).

The exact role of single species floc or aggregate formation is unclear. However, data suggest that these multicellular aggregates may be a defense mechanism adopted by some yeast strains to generate nutritionally rich microenvironments by selective lysis in order to survive such adverse conditions (13). It has also been suggested that they provide the organism with a competitive advantage (14), as studies show that cell-cell adhesion plays a role in self-recognition and the social organization of S. cerevisiae. Thus, adhesion promotes recognition and the physical connection between cells to trigger survival responses, such as alterations to cell wall composition (10, 14).

While the genetic regulation of *FLO* genes in S. cerevisiae has been fairly well elucidated, questions remain regarding the origins and roles of this multigene family with seemingly overlapping, even redundant functions. Given the compact and efficient organization of the S. cerevisiae genome, it is unlikely that the different members of the *FLO* gene family would have been retained from an evolutionary perspective if some unique and critical functions were not imparted by these genes in conditions the yeast would encounter in its natural habitat, a habitat copopulated by numerous other genera and species of yeast.

Furthermore, although the cell-cell adhesion behavior of S. cerevisiae has been widely studied, very little is known regarding the adhesion behavior of other species of yeast. For example, nine genes (named *KaFLO1* to *KaFLO9*) in the yeast Hanseniaspora uvarum were found to contain adhesion-related domains as well as repeated sequences in a study by Pu et al. (15). This suggests that H. uvarum and likely other species of yeast also have a large *FLO* gene family that controls cell-cell adhesion. Genome sequences from other yeast species show that these species also harbor PA14 lectin-binding domains (16).

Rossouw et al. (12) reported that adhesion can occur between different species of yeast and that these interactions show specificity for different combinations of yeast. Moreover, the different members of the *FLO* gene family differentially impact the aggregation outcomes for different pairings of yeast species. While Rossouw et al. (12) investigated the degree to which individual *FLO* genes influence coaggregation between species, here we seek to utilize and explore these attributes further. Indeed, since the differentially expressed *FLO* genes lead to selective aggregation and adhesion, they can be used to evaluate the consequences of physical associations on population dynamics and species fitness in model consortia.

Laboratory yeast strains (genetic background FY23) overexpressing individual *FLO* genes (*FLO1*, *FLO5*, and *FLO11*) under the control of the *HSP30* promoter and three non-Saccharomyces yeast species were used to model the impact of selective aggregation on population outcomes (17). The FY23 strain is a *flo8* deletion mutant that presents a null adhesion background with which to assess the impact of selective yeast-yeast adhesion in the model consortia. The term overexpression as applied to this study simply refers to the controlled expression of *FLO* genes at sufficient levels to induce a consistent adhesion reponse, driven by a single *FLO* gene only under standard laboratory and fermentation conditions. Expressing the *FLO* genes individually in a null background presents the most rational control system, since individually deleting *FLO* genes from an adhesion-competent background would mean that several different *FLO* genes would be expressed together. This expression would obscure any findings linked to specific members of the *FLO* gene family.

The three non-Saccharomyces yeasts, namely Lachancea thermotolerans, Wickerhamomyces anomalus, and Hanseniaspora opuntiae, were selected based on the results of a previous study which showed interesting trends with regard to their interspecies adhesion behavior (12). Importantly, these non-Saccharomyces species are part of the vineyard and wine fermentation ecosystem, and their presence, sometimes in dominant numbers, has been reported in numerous studies (18, 19).

The selective nature of the different members of the *FLO* gene family in terms of interspecies physical aggregation (12) is a useful property that allows for the control and manipulation of interspecies cell-cell contact. Our results show that interspecies contact significantly impacts population dynamics and the survival of individual species in simplified wine-like ecosystems. The data suggest that selective physical interactions between multiple species play a major role in multispecies yeast ecosystem outcomes. While the mechanistic basis for these outcomes is not clear, the impact of differential physical aggregation is clear and pronounced.

## RESULTS

### Pairwise strain interactions.

**FIG 1:**
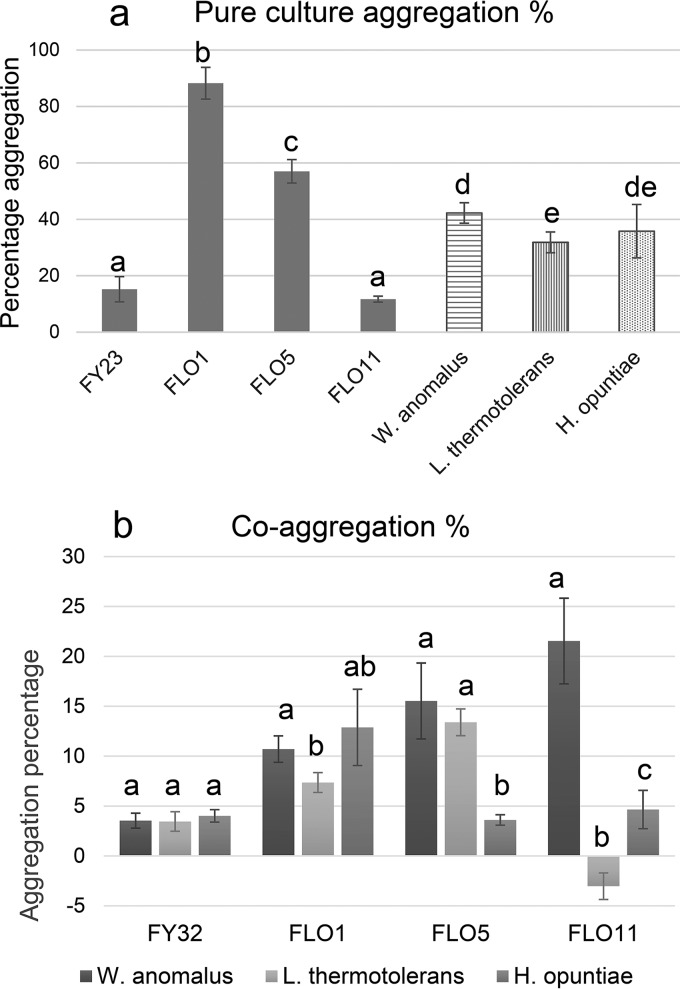
(a) Pure culture aggregation of strains used in this study, (b) as well as coaggregation of the three non-*Saccharomyces* yeasts in combination with each of the *FLO*overexpressing strains and control FY23. All values are the average of five repeats ± standard deviation. (a) Lowercase letters indicate significant differences (*P* < 0.05) between all strains in pure culture and (b) between the aggregation percentages of the non-*Saccharomyces* yeasts for each of the *FLO* treatments separately.

Individual or pure-culture adhesion of the selected non-Saccharomyces yeast strains as well as their coaggregation with the S. cerevisiae mutant strains (the percent increase or decrease in adhesion in coculture compared with the respective individual pure cultures) were assessed in yeast nitrogen base (YNB) cultures, and the results are shown in Fig. 1.

The pure-culture aggregation, percentages of these strains are aligned with the results of previous studies (12, 17), while all three non-Saccharomyces yeasts sediment at higher levels than the unmodified S. cerevisiae strain (Fig. 1a). W. anomalus yeast has the highest sedimentation percentage of 40% in pure culture. All three *FLO*-overexpressing strains coaggregate with W. anomalus (Fig. 1b), while only the *FLO1*-overexpressing strain coaggregates with H. opuntiae. Both the *FLO1* and *FLO5* strains coaggregate with L. thermotolerans, although *FLO11* does not. The negative coaggregation percent shown for the *FLO11*-L. thermotolerans coculture means that the degree of adhesion and sedimentation of these species combined is less than would be expected based on the individual pure culture sedimentation percentages of the two strains.

**FIG 2:**
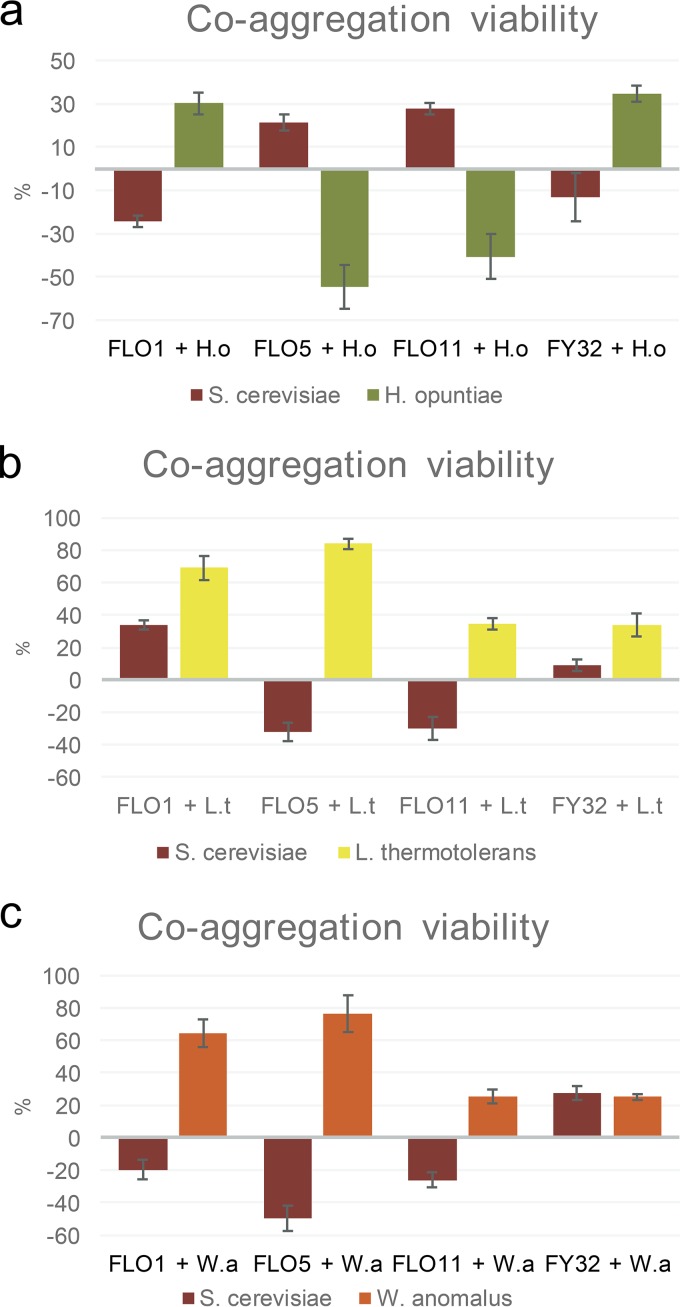
Percent increase (or decrease) in 24-h survival of individual species grown under aggregating conditions compared with nonaggregating conditions. Pairwise combinations were set up between the three overexpressing strains and control FY23 and each non-*Saccharomyces* yeast, namely (a) *H. opuntiae* (H.o), (b) *L. thermotolerans* (L.t), and (c) *W. anomalus* (W.a). Data for *S. cerevisiae* are indicated by red bars and for the non-*Saccharomyces* yeast by green, yellow, and orange bars for *H. opuntiae*, *L. thermotolerans*, and *W. anomalus* respectively. *P* values are shown in Table S1.

To investigate the impact of this selective adhesion in pairwise combinations on the survival of one or both partners, pairs of assays were set up to compare cell viability after 16 h in saline solution containing either no (nonadhesive conditions) or a small amount of CaCl_2_ to induce coaggregation. Figure 2 shows the impact (percent increase or decrease) on cell surivival for both species in the coaggregating cultures compared with that of the nonaggregating cell suspensions where no cell-cell adhesion occurs. The percent increase or decrease in the viability of the S. cerevisiae or non-Saccharomyces yeast strains is shown for the aggregating conditions (adhesion-inducing) relative to nonaggregating conditions. While this system is oversimplified and does not reflect the complexity of a natural system, it provides a means to assess the direct impact of interspecies adhesion without confounding factors, such as competition for nutrients influencing the outcomes, and is in scope equivalent to the previously published data based on membrane bioreactors.

10.1128/mSphere.00383-18.1 TABLE S1 *P* values for pairwise S. cerevisiae and non-Saccharomyces yeast (L. thermotolerans, H. opuntiae, or W. anomalus) comparisons of viability percents after 24 h of two-species coculture (data shown in Fig. 2). Download Table S1, DOCX file, 0.02 MB. Copyright © 2018 Rossouw et al. 2018 Rossouw et al. This content is distributed under the terms of the Creative Commons Attribution 4.0 International license.

Under aggregating conditions, the *FLO5* and *FLO11* strains (which do not coaggregate with H. opuntiae) showed improved survival (20% to 25% greater number of viable cells) compared with the S. cerevisiae in the parallel nonaggregating conditions. This suggests that S. cerevisiae benefits by self-adhesion under these circumstances (Fig. 2a). Keeping in mind that flo11p does not have a PA14 lectin domain, the cell-cell interactions between the *FLO11*-expressing S. cerevisiae and non-Saccharomyces yeasts may involve lectin-indepedent or mannan-independent aggregation. While the interactions in the case of the *FLO11* treatments may be aspecific, different than *FLO1* and *FLO5* in this regard, the adhesion outcomes are nonetheless different for different species combinations, regardless of whether this is due to the direct action of the Flo11p.

On the other hand, coaggregation between the *FLO1* and *H.* opuntiae strains is detrimental to S. cerevisiae, leading to a 20% lower viability of S. cerevisiae under these conditions than the same cultures in nonaggregating conditions. In contrast, direct physical contact between the *FLO1* strain of S. cerevisiae and H. opuntiae, while detrimental to S. cerevisiae, provides an advantage of some sort to the H. opuntiae in terms of survival, as the H. opuntiae yeasts in the coaggregating mixed cultures show, on average, a 30% greater viability than the same treatment in nonaggregating conditions. This indicates that the effect of the interaction on cell viability is based on direct and sustained physical contact between the different species, contrasted to the nonaggreating conditions where the two cocultured species are only able to interact transiently with one another.

In contrast, a very different trend is observed in the presence of L. thermotolerans yeasts, where the S. cerevisiae *FLO5* and *FLO11* strains have decreased survival (greater than 30% decline) under aggregating conditions compared with the same mixed cultures grown under nonaggregating conditions. The L. thermotolerans strain, on the other hand, shows an increase in survival of up to 80% in the coaggregating cultures compared with nonaggregating conditions for the paired cultures (Fig. 2b). Interestingly, coaggregation between the *FLO1*-overexpressing S. cerevisiae and L. thermotolerans strains is beneficial for both parties. This contrasts strongly with the results of the W. anomalus strain pairings (Fig. 2c), where coaggregation between the *FLO*-overexpressing strains and W. anomalus strains is in all cases to the detriment of S. cerevisiae, and the benefit of the W. anomalus. These findings highlight the complexity of physical interactions between different species of yeast and their impact on the growth and viability of the species involved.

### Population dynamics in multispecies consortia.

In order to assess the impact of the *FLO* gene-dependent physical interactions on population outcomes in a model system, a simplified design was implemented using a defined synthetic grape must, reflecting the composition of a grape must after pressing. Pressed grape must is an important environmental niche for industrially relevant fermentation microorganisms. The fermentation environment has arguably played an important role in the domestication and evolution of commercial yeasts (20, 21), an important consideration when evaluating the impact of gene families related to intraspecies and interspecies interactions. This sytem also allows for an extended (more than 2 weeks) period of batch culture and growth and provides the opportunity to observe population dynamics over a longer time course. Different combinations of the four S. cerevisiae strains and the three non-Saccharomyces yeasts (multifactorial three-way pairings, as well as all four species together) were inoculated into the synthetic must to create multispecies systems more representative of the complexity of a natural environment, yet simple enough to monitor and characterize adequately. In each treatment, all strains were inoculated at equal cell densities (CFU ml^−1^). Depending on the particular combination of species, the identity of the *FLO* gene overexpressed, as well as the stage of the fermentation process, significant differences were observed in the composition of the yeast population.

### S. cerevisiae, L. thermotolerans, and H. opuntiae.

**FIG 3:**
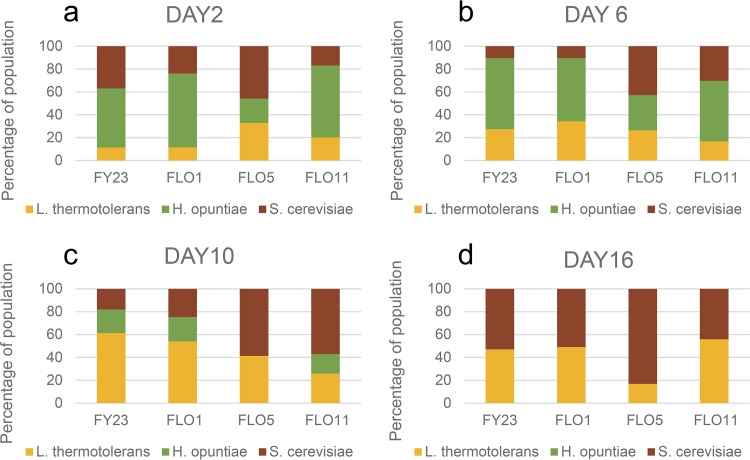
Percent composition of *S. cerevisiae*, *L. thermotolerans*, and *H. opuntiae* in three-species cocultures by days 2 (a), 6 (b), 10 (c), and 16 (d) of fermentative growth. Four parallel sets of cultures were inoculated with either the control FY23 or one of the three *FLO*-overexpressing strains of *S. cerevisiae*. Values are the average of three repeats.

In the absence of W. anomalus, H. opuntiae is generally the dominant species (or codominant) in the early stages of fermentation (Fig. 3a and b). Under these circumstances, coaggregation with H. opuntiae (as the *FLO1* strain and control FY23 do) is to the detriment of S. cerevisiae. However, the *FLO11* and *FLO5* strains do not coaggregate with H. opuntiae and constitute a large proportion (40% and 50%, respectively) of the yeast population by day 6 of growth in these consortia (Fig. 3c). H. opuntiae is present at the lowest levels of all three species in the *FLO5* treatment (Fig. 3c). Coaggregation between the *FLO5*-overexpressing S. cerevisiae and L. thermotolerans strains (Fig. 1b) appears to be advantageous to both species under these conditions, enabling them to outcompete H. opuntiae, compared with *FLO1*, *FLO11*, and the control (Fig. 3c).

In the absence of W. anomalus, the dominant species is S. cerevisiae in the *FLO5* treatment (90%). This is also the only one of the four *FLO* treatments where H. opuntiae is absent (or below detection levels) by day 10 (Fig. 3c). In FY23, *FLO1*, and *FLO11*, S. cerevisiae and L. thermotolerans are present at more or less equal levels by the end of fermentation (Fig. 3d), with H. opuntiae no longer present in any of the treatments.

### S. cerevisiae, L. thermotolerans, and W. anomalus.

**FIG 4:**
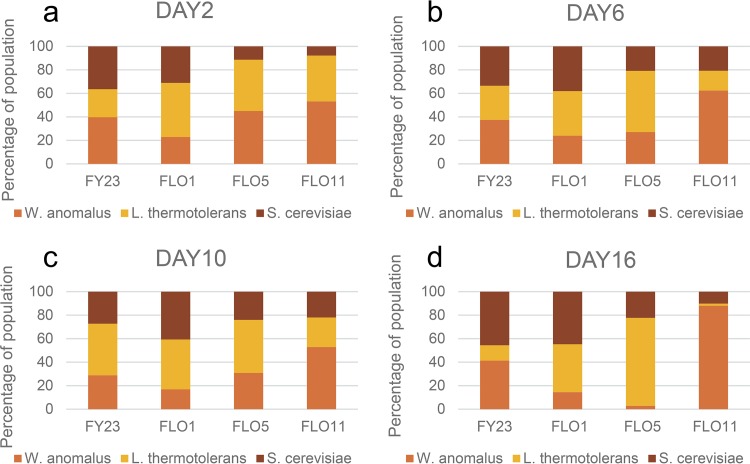
Percent composition of *S. cerevisiae*, *W. anomalus*, and *L. thermotolerans* in three-species cocultures by days 2 (a), 6 (b), 10 (c), and 16 (d) of fermentative growth. Four parallel sets of cultures were inoculated with either the control FY23 or one of the three *FLO*-overexpressing strains of *S. cerevisiae*. Values are the average of three repeats.

In strain combinations that include W. anomalus, different dynamics can be observed. In the absence of H. opuntiae (Fig. 4a to d), S. cerevisiae in the *FLO5* and *FLO11* treatments show the lowest survival (20% and 10% abundance, respectively, by day 16), while in the *FLO1* treatment, S. cerevisiae consitutes 40% of the total population and W. anomalus constitutes less than 20%. In contrast, W. anomalus is the dominant species in the *FLO11* treatment, both at day 2 and day 6 (Fig. 4a and b). Interestingly, while the levels of S. cerevisiae in the *FLO5* and *FLO11* treatments are similar at day 6 (Fig. 4b), the dominant species (at 50%) in the *FLO5* treatment is L. thermotolerans, contrasted with the *FLO11* treatment where W. anomalus is dominant (60%).

In the absence of H. opuntiae, coaggregation between *FLO1* and L. thermotolerans appears to benefit both parties, enabling them to outcompete W. anomalus, compared with the control FY32 and *FLO11* treatment (Fig. 4a to d). Although *FLO5* does coaggregate with L. thermotolerans (Fig. 1b), this appears to be to the detriment of the S. cerevisiae but to the benefit of L. thermotolerans, which is able to outcompete W. anomalus (compared with the control strain) in the early stages and outcompete S. cerevisiae in the later growth stages (Fig. 4c and d). This broadly aligns with the results of the pairwise inhibition/viability assays (Fig. 2b), which pointed toward a mutually beneficial association between the *FLO1*-overexpressing S. cerevisiae and L. thermotolerans.

### S. cerevisiae, H. opuntiae, and W. anomalus.

**FIG 5:**
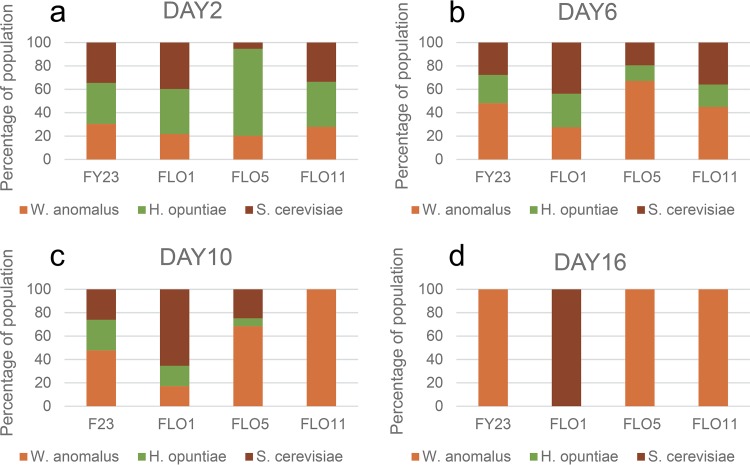
Percent composition of *S. cerevisiae*, *W. anomalus*, and *H. opuntiae* in three-species cocultures by days 2 (a), 6 (b), 10 (c), and 16 (d) of fermentative growth. Four parallel sets of cultures were inoculated with either the control FY23 or one of the three *FLO*-overexpressing strains of *S. cerevisiae*. Values are the average of three repeats.

When W. anomalus and H. opuntiae are present in consortia lacking L. thermotolerans, the proportion of H. opuntiae and S. cerevisiae in the populations of these two treatments decline as W. anomalus increases by day 6 of growth (Fig. 5b). The exception is the *FLO1* treatment, where S. cerevisiae and H. opuntiae are codominant at approximately 40% and 30% for days 2 and 6, respectively (Fig. 5a and b). The results for *FLO1*-overexpressing S. cerevisiae are the most starkly contrasted with the control and other treatments: When L. thermotolerans is not present in the ecosystem, W. anomalus dominates the fermentation in all treatments (at 100%), but for the *FLO1*-overexpressing treatment, it is S. cerevisiae which is dominant at 100% abundance by day 16 (Fig. 5c and d).

This strongly suggests that *FLO1*-mediated physical interaction provides a competitive advantage over W. anomalus in a scenario where other strong competitors (such as L. thermotolerans) are absent. Coaggregation between the *FLO1* strain and H. opuntiae in the early stages of fermentation may have provided the S. cerevisiae in this treatment with an ally initially to restrict the growth of the W. anomalus. In support of this speculative federation, H. opuntiae is still present in the *FLO1* treatment by day 10 of fermentation in significant amounts but has all but disappeared in the *FLO5* and *FLO11* treatments.

### Four species consortia.

**FIG 6:**
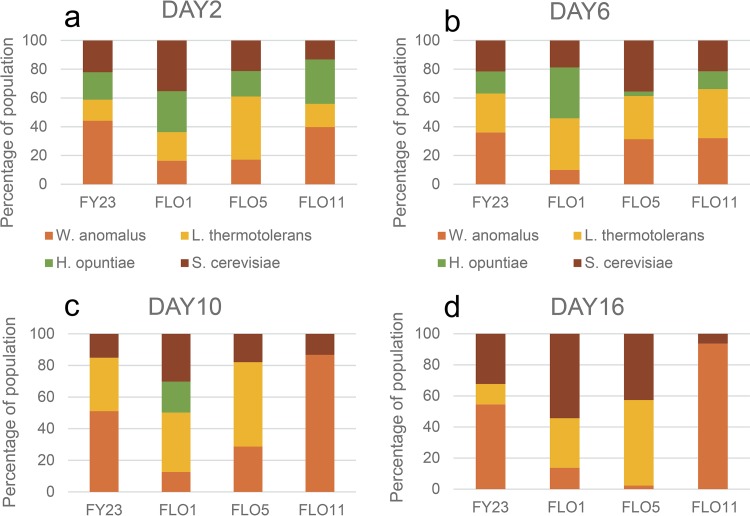
Percent composition of *S. cerevisiae*, *W. anomalus*, *L. thermotolerans*, and *H. opuntiae* in four-species cocultures at days 2 (a), 6 (b), 10 (c), and 16 (d) of fermentative growth. Four parallel sets of cultures were inoculated with either the control FY23 or one of the three *FLO-*overexpressing strains of *S. cerevisiae*. Values are the average of three repeats.

S. cerevisiae and L. thermotolerans are the dominant species in the *FLO1* treatments by day 10 (Fig. 6c), with S. cerevisiae dominating at more than 50% of the total population by day 15 (Fig. 6d). At this point, very little (less than 15%) W. anomalus is present. However, W. anomalus is the dominant species toward and at the end of fermentative growth for the *FY23* and *FLO11* treatments, almost exclusively so in the case of *FLO11* (Fig. 6d). The *FLO5*-overexpressing S. cerevisiae shows a greater percent contribution to the overall population (40%) than the control and *FLO11* strain; however, in this case, L. thermotolerans is the dominant species by day 16 (Fig. 6d). In all *FLO5* treatments containing W. anomalus, the dominant species is W. anomalus in the absence of L. thermotolerans, but L. thermotolerans dominates when both W. anomalus and L. thermotolerans are present in consortia (Fig. 3 to 6).

In general, the *FLO1* and *FLO5* strains appear to follow different strategies for collegial associations with other strains at different stages of fermentation. Both strategies are successful (to a greater or lesser degree), as S. cerevisiae levels for *FLO1* and *FLO5* are higher at most stages of fermentative growth than the control for the 4-strain treatment. However, the relative levels of the other three species are strongly influenced by the identity of the overexpressed *FLO* gene.

## DISCUSSION

Although the number of species and strains used in our simplified consortia does not represent the natural complexity of most yeast ecosystems, the results highlight the dramatic impact of differential physical interaction (as mediated by different members of the FLO adhesion protein family), compared with noninteracting yeasts, on population dynamics and survival. Importantly, while the data are based on a highly simplified ecosystem in well-controlled environments, the inoculation ratios and cell concentrations of our simplified consortia are within the range in which these species are sometimes encountered in spontaneously fermenting grape juice, for example.

While it has been reported that *FLO* genes cause differential adhesion between Saccharomyces cerevisiae and specific species of non-Saccharomyces yeast (12), here we show that these different adhesion relationships have very significant consequences for the balance of species in the ecosystem. The results show that, in this controlled model system, *FLO5* overexpression aligns S. cerevisiae with L. thermotolerans, providing it with an advantage in competition with W. anomalus. This strain does not coaggregate with H. opuntiae and in the absence of L. thermotolerans is rapidly outcompeted by W. anomalus. Although the *FLO5* strain does coaggregate with W. anomalus, this appears to be to its detriment.

The *FLO1*-overexpressing S. cerevisiae, in contrast, coaggregates with H. opuntiae, which appears to be a beneficial association in the early stages of fermentation, providing the *FLO1* strain with an advantage compared with the other three S. cerevisiae strains (particularly in the presence of W. anomalus). In the H. opuntiae-containing treatments paired with the *FLO1*-overexpressing strain, the H. opuntiae also persists to later stages of fermentation compared with the other strain treatments.

W. anomalus rapidly outcompetes the *FLO11*-overexpressing S. cerevisiae strain in all treatments where it is present, as *FLO11* does not coaggregate with either H. opuntiae or L. thermotolerans. Partnering with one of these strains (H. opuntiae in early fermentation or L. thermotolerans in later stages of fermentation) appears to be necessary to mount a defensive against the otherwise dominant W. anomalus (or simply to reduce physical interactions with W. anomalus, which appears inhibitory to S. cerevisiae).

Considered together, the results clearly show that different members of the *FLO* gene family exert a notable influence in terms of yeast species demographics at different stages of fermentation in a model system. While these genes only mediate adhesion, the resulting physical interaction leads to a species-specific growth response, mediated by mechanisms and means yet to be elucidated. Considering that intermicrobial interactions constitute one of the main selection pressures in natural ecosystems, it is reasonable to speculate that the *FLO* gene family (the only gene family responsible for asexual adhesion) may play a role in the evolution of cooperativity and antagonism between different species of yeast.

In support of this theory, The *FLO1*, *FLO5*, *FLO9*, and *FLO10* genes are carried in subtelomeric loci (22), which holds important implications for the evolution of *FLO* genes, as subtelometric loci are subject to increased recombination frequencies (23). In addition, *FLO* genes contain up to 20 tandemly repeated sequences in their middle region which can lead to high mutation frequencies by recombination events (24, 25). The frequent recombination of *FLO* genes is thought to be an important mechanism for the rapid adaptation of adhesion properties of natural yeast in changing environments (26). Indeed, it has been shown that the *FLO* gene family has evolved and expanded extraordinarily fast (27). Both interchromosomal and intrachromosomal ectopic recombination are considered to occur for *FLO* gene paralogs (28, 29). More specifically, two types of recombination events occur between *FLO* genes. First, recombination events occur across small regions of homology in the N-terminal or C-terminal domain of *FLO* genes. Recombination events in the N-terminal can alter the strength and preference of substrate binding and hold implications for the function of modified *FLO* genes. Second, recombination across the central repeat domains of *FLO* genes leads to variation in the length and sequence of the repeat regions (28).

Understanding the molecular mechanisms and regulation of interspecies adhesion processes, as well as the impacts thereof on interspecies interaction dynamics, is important in terms of potential industrial application. Considering that cell-cell adhesion appears to play a pivotal role in the survival and social dynamics of yeast populations in natural environments, this information is also important to our understanding of possible evolutionary mechanisms linked to physical interactions between different microorganisms in shared ecological niches.

Evolutionary studies have clearly demonstarted that S. cerevisiae has undergone significant evolutionary changes, sometimes referred to as “domestication” due to the opportunities provided by human-made fermentation environments (20, 21) which would include adaptations that favor its relative fitness in these multispecies fermentation ecosystems.

To the best of our knowledge, no other gene family has yet shown such dramatic effects on population dynamics in multispecies systems. The data clearly demonstrate that the assortment of *FLO* genes at the disposition of S. cerevisiae would allow this yeast to selectively adapt to challenges presented by differing and rapidly changing yeast-rich environmental niches. Indeed, it can be argued that no other genetic system in S. cerevisiae would provide the same type of flexibility, responsiveness, and advantages for rapid adaptation. The large number of *FLO* genes, mostly located in subtelomeric recombination hotspots, combined with epigenetic regulation allowing for population-wide adjusted switches of *FLO* gene expression, would allow for rapid adjustment to the challenges of interspecies competition in changing yeast ecosystems. Considering that S. cerevisiae is present at less than 1% (sometimes even undetectable levels) of the yeast population at the start of fermentation, selective associations with dominant yeast species could provide S. cerevisiae with an advantage in the initial fermentation stages.

Future work should seek to investigate the genetics and expression of *FLO*-equivalent adhesion genes in species of non-Saccharomyces yeast, focusing in particular on the impacts which different species combinations have on the expression of these genes as population dynamics evolve over time. In addition, the role of the members of the *FLO* gene family in multispecies biofilms should be investigated, given the importance of biofilm formation in microbial persistence in humans and hospital equipment (30).

## MATERIALS AND METHODS

### Strains, media, and culture conditions.

The yeast strains used in this study were selected from the strain collection at the Institute for Wine Biotechnology (Table 1). The S. cerevisiae strains used are described by Govender et al. (17). They include the FY23 laboratory strain which is nonflocculent due to a mutation in the *FLO8* gene, as well as three strains each overexpressing one *FLO* gene, namely *FLO1*, *FLO5*, and *FLO11*, under the control of the HSP30 promoter construct, which is induced at the onset of stationary phase as well as under certain stresses, such as heat shock (17). The non-Saccharomyces yeast strains used were Wickerhamomyces anomalus, Lachancea thermotolerans, and Hanseniaspora opuntiae, which were previously described in Rossouw et al. (12). Strains were maintained on YPD agar from pure frozen cultures. Liquid overnight cultures were grown in 5 ml YPD broth (BioLab, South Africa) to exponential phase at 30°C. Wallerstein nutrient (WLN) agar (BioLab) was used for culturing and enumerating yeast from fermentations and assays.

**TABLE 1:** *S. cerevisiae* overexpression strains and non-*Saccharomyces* strains used in this study (12, 16)

Species	Strain or isolate	Genotype
Saccharomyces cerevisiae	FY23	*MATa leu2 trp1 ura3 flo8-*1
	FY23-F1H	*MATa leu2 trp1 ura3 flo8-1 FLO1p*::*SMR1-HSP30p*
	FY23-F5H	*MATa leu2 trp1 ura3 flo8-1 FLO5p*::*SMR1-HSP30p*
	FY23-F11H	*MATa leu2 trp1 ura3 flo8-1 FL*O11p::*SMR1-HSP30p*
Wickerhamomyces anomalus	IWBT-Y934	
Lachancea thermotolerans	IWBT-Y983	
Hanseniaspora opuntiae	IWBT-Y1055	

### Ca^2+^-dependent aggregation assays.

To quantify the degree to which individual strains aggregate, flocculation assays were carried out as described previously (6, 7, 17). Since FLO lectin-dependent aggregation only occurs in the presence of Ca^2+^, these assays are based on measuring the optical density of cell suspensions before and after the addition of Ca^2+^. Greater differences in the optical densities before and after Ca^2+^ addition reflect greater aggregation and sedimentation rates, and vice versa. Initially, yeast colonies for each isolate were inoculated (6 repeats) in test tubes containing 5 ml soyabean casein digest (SCD) medium and grown to stationary phase. An aqueous solution of EDTA (pH 8.0) was added to these cultures to a final concentration of 50 mM, and the cultures were agitated vigorously by vortexing at the maximum speed setting. The optical density at 600 nm (OD_600_) was determined immediately (reading A). Ca^2+^-dependent aggregation was subsequently induced by spinning down 1 ml of the liquid cultures in a microcentrifuge, followed by washing in 1 ml ddH_2_O and resuspension in 1 ml of 40 mM CaCl_2_. The samples were then vigorously agitated as before and left undisturbed for 60 s. A sample was taken from below the meniscus in the microcentrifuge tube of each sample and mixed thoroughly with 160 µl of a 40 mM CaCl_2_ solution. A second spectrophotometric measurement was then taken at a wavelength of 600 nm as before (reading B). For more information see Bester et al. (7). The extent of Ca^2+^-dependent aggregation was then calculated using the following formula:

$$Aggregation\;\left(\%\right)=\frac{A-B}{A}\;\times \;100$$

To calculate the extent of coaggregation between different species of yeast in mixed cultures, S. cerevisiae strains and the non-Saccharomyces yeasts under investigation were combined in a 1:1 cell:cell ratio and the assay carried out using the mixed culture as described in the preceding section. The total cell concentrations in the coaggregation assays (i.e., S. cerevisiae plus non-Saccharomyces strain) were the same as for pure cultures. The aggregation percent was calculated as before, and the coaggregation percent was calculated by subtracting the expected aggregation rate (based on the combined average percentages of the pure cultures) from the experimentally determined aggregation percent obtained for the combined cultures.

### Microscopy.

Alexa Fluor wheat germ agglutinin (WGA) conjugate (Invitrogen) staining of cells and fluorescence microscopy were carried out as described by Wright (31). Image acquisition was performed on an Olympus cell system attached to an IX 81 inverted fluorescence microscope equipped with an F-view II cooled CCD camera (Soft Imaging Systems). The excitation lasers used were the 495-nm wavelength for WGA 488 (green) and 679 nm for WGA 680 (red), and the emission filters used were 519 nm and 702 nm, respectively. Images were processed and background subtracted using the Cell software and presented in a maximum intensity projection. Cell cultures were combined in a 1:1 ratio of the non-Saccharomyces yeast under investigation in combination with each of the *FLO* gene-overexpressing strains and control FY32 separately (1 × 10^7^ cells/ml of each). Species were individually prestained (S. cerevisiae in red, non-Saccharomyces yeast in red) and the species combined under aggregating (containing Ca^2+^) and nonaggregating (no Ca^2+^, containing EDTA) conditions. Samples of cell sediments were taken for microscopic evaluation as described.

### Pairwise interaction assays.

The three non-Saccharomyces strains and overexpression strains (Table 1) were washed three times (after preculture in YPD) and coinoculated into buffered saline solution containing either 5 mM EDTA (nonaggregating conditions) or 10 mM CaCl_2_ (inducing Flo protein-driven aggregation). Culturing paired species with and without CaCl_2_ allows for the determination of cell survival of both species after 16 h when in direct physical contact in multispecies aggregates. After 16 h, serial dilutions were plated onto WLN agar to allow for differential identification and quantification (CFU ml^−1^) of the S. cerevisiae and W. anomalus, L. thermotolerans, and H.opuntiae in the various pairings under coaggregating versus nonaggregating conditions. Interaction assays were performed in quadruplicate. The percent increase/decrease of the yeast species in these assays was calculated under the aggregating conditions relative to the nonaggregating conditions.

### Multispecies growth experiments.

Cells were inoculated and grown in a chemically defined synthetic must under fermentative conditions, mimicking a natural environment for multispecies yeast communities. These growth conditions allow for an extended growth period and observation window for the yeast-yeast interactions over time, compared with those of conventional rich medium and aerobic growth conditions. The medium used is based on the formulation of the Australian Wine Research Institute (32), with amino acid additions as described by Bely et al. (33). Sugar concentrations were 100 g/liter each of glucose and fructose, and the pH of the medium was adjusted to 3.3 with NaOH. Strains were precultured onto YPD and coinoculated in 80 ml fermentation flasks at an OD_600_ of 0.1 each. The following combinations were used:

1. L. thermotolerans, W. anomalus, S. cerevisiae (FY23/*FLO1*/*FLO5*/*FL*O11)
2. L. thermotolerans, H. opuntiae, S. cerevisiae (FY23/*FLO1*/*FLO5*/*FL*O11)
3. W. anomalus, H. opuntiae, S. cerevisiae (FY23/*FLO1*/*FLO5*/*FL*O11)
4. L. thermotolerans, W. anomalus, H. opuntiae, S. cerevisiae (FY23/*FLO1*/*FLO5*/*FL*O11)

All treatments were carried out in triplicate. Samples were taken at days 1, 2, 5, 10, and 16 (the end of alcoholic fermentation) for analysis of sugars and for DNA extraction.

### Automated ribosomal intergenic spacer analysis.

DNA extraction was carried out on samples taken from the multispecies fermentations as described by Hoffman (34). Automated ribosomal intergenic spacer analysis (ARISA) was subsequently performed using 50 ng of DNA template and carboxy-fluorescein-labeled forward (ITS1-6FAM) and ITS4 primers (35, 36). The labeled PCR products were separated by capillary electrophoresis on an ABI 3,010 × I Genetic analyzer (Applied Biosystems) at the Central Analytical Facility, Stellenbosch University. The raw data were converted to electropherograms and further analyzed in Genemapper 4.1 (Applied Biosystems). Peak areas for each species in the consortium as well as S. cerevisiae were calculated to determine the relative species abundance in each fraction. The average abundance of each of the individual peaks was calculated and represented as a percentage of the total number of peak heights displayed in each sample. Statistical analyses were conducted using XLStat 2017.[Supplementary-material tabS2][Supplementary-material tabS3][Supplementary-material tabS4][Supplementary-material tabS5]

10.1128/mSphere.00383-18.2 TABLE S2 Significance values for different *FLO* treatments in S. cerevisiae, L. thermotolerans, and H. opuntia cocultures at days 2, 6, 10, and 16 of growth. Download Table S2, DOCX file, 0.02 MB. Copyright © 2018 Rossouw et al. 2018 Rossouw et al. This content is distributed under the terms of the Creative Commons Attribution 4.0 International license.

10.1128/mSphere.00383-18.3 TABLE S3 Significance values for different *FLO* treatments in S. cerevisiae, L. thermotolerans, and W. anomalus cocultures at days 2, 6, 10, and 16 of growth. Download Table S3, DOCX file, 0.02 MB. Copyright © 2018 Rossouw et al. 2018 Rossouw et al. This content is distributed under the terms of the Creative Commons Attribution 4.0 International license.

10.1128/mSphere.00383-18.4 TABLE S4 Significance values for different *FLO* treatments in S. cerevisiae, H. opuntiae, and W. anomalus cocultures at days 2, 6, 10, and 16 of growth. Download Table S4, DOCX file, 0.02 MB. Copyright © 2018 Rossouw et al. 2018 Rossouw et al. This content is distributed under the terms of the Creative Commons Attribution 4.0 International license.

10.1128/mSphere.00383-18.5 TABLE S5 Significance values for different *FLO* treatments in S. cerevisiae, H. opuntiae, L. thermotolerans, and W. anomalus cocultures at days 2, 6, 10, and 16 of growth. Download Table S5, DOCX file, 0.02 MB. Copyright © 2018 Rossouw et al. 2018 Rossouw et al. This content is distributed under the terms of the Creative Commons Attribution 4.0 International license.
