# Supplementary material for: Modifying Saccharomyces cerevisiae Adhesion Properties Regulates Yeast Ecosystem Dynamics
Source: mSphere. 2018 Oct 24;3(5):e00383-18. doi: 10.1128/mSphere.00383-18 (PMC6200983; doi:10.1128/mSphere.00383-18)
Supplement: Supplementary file 1 [file sph005182668st1.docx]

S. cerevisiae + H. opuntiae

	S. cerevisiae				*H. opuntiae*			
	FY23	FLO1	FLO5	FLO11	FY23	FLO1	FLO5	FLO11
FY23	0	0.227387638	0.023735	0.019433	0	0.30987	0.0016244	0.002766
FLO1	0.227388	0	0.000201	1.66E-05	0.30987	0	0.0010984	0.002076
FLO5	0.023735	0.000200852	0	0.083482	0.001624	0.001098	0	0.169422
FLO11	0.019433	1.66281E-05	0.083482	0	0.002766	0.002076	0.1694219	0

S. cerevisiae + L. thermotolerans

	S. cerevisiae				*L. thermotolerans*			
	FY23	FLO1	FLO5	FLO11	FY23	FLO1	FLO5	FLO11
FY23	0	0.00077	0.00113	0.00424	0	0.00402	0.00200	0.90905
FLO1	0.00077	0	0.00050	0.00173	0.00402	0	0.05602	0.00679
FLO5	0.00113	0.00050	0	0.69372	0.00200	0.05602	0	0.00005
FLO11	0.00424	0.00173	0.69372	0	0.90905	0.00679	0.00005	0

S. cerevisiae + W. anomalus

	S. cerevisiae				*W. anomalus*			
	FY23	FLO1	FLO5	FLO11	FY23	FLO1	FLO5	FLO11
FY23	0	0.00068	0.00053	0.00012	0	0.01350	0.01415	0.91459
FLO1	0.00068	0	0.00693	0.21607	0.01350	0	0.20916	0.00662
FLO5	0.00053	0.00693	0	0.01633	0.01415	0.20916	0	0.00878
FLO11	0.00012	0.21607	0.01633	0	0.91459	0.00662	0.00878	0
