# Supplementary material for: Modifying Saccharomyces cerevisiae Adhesion Properties Regulates Yeast Ecosystem Dynamics
Source: mSphere. 2018 Oct 24;3(5):e00383-18. doi: 10.1128/mSphere.00383-18 (PMC6200983; doi:10.1128/mSphere.00383-18)
Supplement: Supplementary file 2 [file sph005182668st2.docx]

		L. thermotolerans				*H. opuntiae*				*S. cerevisiae*			
		**FY23**	FLO1	FLO5	FLO11	**FY23**	FLO1	FLO5	FLO11	**FY23**	FLO1	FLO5	FLO11
DAY 2	**FY23**	**0**	0.722339	**0.00972**	**0.008363**	**0**	0.101304	**0.000701**	**0.010944**	**0**	0.072884	0.07267	**0.003217**
	FLO1	0.722339	**0**	**0.012825**	**0.008909**	0.101304	**0**	**0.031057**	0.602345	0.072884	**0**	**0.011981**	0.124348
	FLO5	**0.00972**	**0.012825**	**0**	**0.020172**	**0.000701**	**0.031057**	**0**	**0.00039**	0.07267	**0.011981**	**0**	**0.010316**
	FLO11	**0.008363**	**0.008909**	**0.020172**	**0**	**0.010944**	0.602345	**0.00039**	**0**	**0.003217**	0.124348	**0.010316**	**0**
Day 6	**FY23**	**0**	0.095094	0.693603	0.094615	**0**	0.069552	**0.012007**	0.300428	**0**	0.981617	**0.010741**	0.060484
	FLO1	0.095094	**0**	0.10223	0.074116	0.069552	**0**	**0.042129**	0.701696	0.981617	**0**	**0.019058**	**0.042802**
	FLO5	0.693603	0.10223	**0**	0.105198	**0.012007**	**0.042129**	**0**	0.106159	**0.010741**	**0.019058**	**0**	0.115965
	FLO11	0.094615	0.074116	0.105198	**0**	0.300428	0.701696	0.106159	**0**	0.060484	**0.042802**	0.115965	**0**
Day 10	**FY23**	**0**	0.061663	**0.031984**	**0.011635**	**0**	0.76714	0.052174	0.288012	**0**	**0.006322**	**0.000173**	**0.00018**
	FLO1	0.061663	**0**	**0.017818**	**0.00255**	0.76714	**0**	**0.014138**	0.055608	**0.006322**	**0**	**0.000179**	**0.000233**
	FLO5	**0.031984**	**0.017818**	**0**	**0.002657**	0.052174	**0.014138**	**0**	**0.005024**	**0.000173**	**0.000179**	**0**	0.073913
	FLO11	**0.011635**	**0.00255**	**0.002657**	**0**	0.288012	0.055608	**0.005024**	**0**	**0.00018**	**0.000233**	0.073913	**0**
Day 16	**FY23**	**0**	0.071264	**0.020403**	**0.047166**	**0**	na	na	na	**0**	0.071264	**0.020403**	**0.047166**
	FLO1	0.071264	**0**	**0.013249**	**0.03851**	na	**0**	na	na	0.071264	**0**	**0.013249**	**0.03851**
	FLO5	**0.020403**	**0.013249**	**0**	**0.001838**	na	na	**0**	na	**0.020403**	**0.013249**	**0**	**0.001838**
	FLO11	**0.047166**	**0.03851**	**0.001838**	**0**	na	na	na	**0**	**0.047166**	**0.03851**	**0.001838**	**0**
