# Supplementary material for: Modifying Saccharomyces cerevisiae Adhesion Properties Regulates Yeast Ecosystem Dynamics
Source: mSphere. 2018 Oct 24;3(5):e00383-18. doi: 10.1128/mSphere.00383-18 (PMC6200983; doi:10.1128/mSphere.00383-18)
Supplement: Supplementary file 3 [file sph005182668st3.docx]

		L. thermotolerans				*W. anomalus*				*S. cerevisiae*			
		**FY23**	FLO1	FLO5	FLO11	**FY23**	FLO1	FLO5	FLO11	**FY23**	FLO1	FLO5	FLO11
DAY 2	**FY23**	**0**	**0.026588**	**0.01781**	0.053949066	**0**	**0.0489**	0.121864	**0.023082**	**0**	0.121555	**0.009196**	**0.034471**
	FLO1	**0.026588**	**0**	0.212905	0.058180822	**0.0489**	**0**	**0.024555**	**0.019306**	0.121555	**0**	**0.016239**	**0.034265**
	FLO5	**0.01781**	0.212905	**0**	0.127247901	0.121864	**0.024555**	**0**	**0.043824**	**0.009196**	**0.016239**	**0**	0.299078
	FLO11	0.053949	0.058181	0.127248	**0**	**0.023082**	**0.019306**	**0.043824**	**0**	**0.034471**	**0.034265**	0.299078	**0**
Day 6	**FY23**	**0**	**0.045607**	**0.006876**	0.053320278	**0**	**0.045441**	0.103782	**0.012634**	**0**	**0.037412**	**0.005655**	**0.019778**
	FLO1	**0.045607**	**0**	**0.028547**	**0.011553896**	**0.045441**	**0**	0.289496	**0.003259**	**0.037412**	**0**	**0.00305**	**0.008702**
	FLO5	**0.006876**	**0.028547**	**0**	**0.005173755**	0.103782	0.289496	**0**	**0.019294**	**0.005655**	**0.00305**	**0**	0.954278
	FLO11	0.05332	**0.011554**	**0.005174**	**0**	**0.012634**	**0.003259**	**0.019294**	**0**	**0.019778**	**0.008702**	0.954278	**0**
Day 10	**FY23**	**0**	0.16987	0.679506	**0.009500552**	**0**	**0.015817**	0.510756	**0.027106**	**0**	**0.024662**	0.242347	0.11892
	FLO1	0.16987	**0**	0.451161	**0.003381155**	**0.015817**	**0**	0.053499	**0.018085**	**0.024662**	**0**	0.057699	**0.033219**
	FLO5	0.679506	0.451161	**0**	0.055659555	0.510756	0.053499	**0**	0.061375	0.242347	0.057699	**0**	0.150886
	FLO11	**0.009501**	**0.003381**	0.05566	**0**	**0.027106**	**0.018085**	0.061375	**0**	0.11892	**0.033219**	0.150886	**0**
Day 16	**FY23**	**0**	**0.007394**	**0.000442**	**0.004656642**	**0**	**0.002387**	**0.004347**	**0.001202**	**0**	0.673075	**0.005304**	**0.002034**
	FLO1	**0.007394**	**0**	**0.001293**	**0.001104558**	**0.002387**	**0**	**0.021598**	**0.000526**	0.673075	**0**	**0.014669**	**0.006071**
	FLO5	**0.000442**	**0.001293**	**0**	**3.39669E-05**	**0.004347**	**0.021598**	**0**	**0.002845**	**0.005304**	**0.014669**	**0**	**0.008862**
	FLO11	**0.004657**	**0.001105**	**3.4E-05**	**0**	**0.001202**	**0.000526**	**0.002845**	**0**	**0.002034**	**0.006071**	**0.008862**	**0**
