# Supplementary material for: Modifying Saccharomyces cerevisiae Adhesion Properties Regulates Yeast Ecosystem Dynamics
Source: mSphere. 2018 Oct 24;3(5):e00383-18. doi: 10.1128/mSphere.00383-18 (PMC6200983; doi:10.1128/mSphere.00383-18)
Supplement: Supplementary file 4 [file sph005182668st4.docx]

		W. anomalus				*S. cerevisiae*				*H. opuntiae*			
		**FY23**	FLO1	FLO5	FLO11	**FY23**	FLO1	FLO5	FLO11	**FY23**	FLO1	FLO5	FLO11
DAY 2	**FY23**	**0**	0.074623	0.061682	0.219028	**0**	0.138059	**0.019183**	0.588757	**0**	**0.018982**	**0.00122**	0.085814
	FLO1	0.074623	**0**	**0.012245**	**0.042957**	0.138059	**0**	**0.000238**	**0.005401**	**0.018982**	**0**	**0.000409**	0.987653
	FLO5	0.061682	**0.012245**	**0**	**0.029439**	**0.019183**	**0.000238**	**0**	**0.001821**	**0.00122**	**0.000409**	**0**	**0.000795**
	FLO11	0.219028	**0.042957**	**0.029439**	**0**	0.588757	**0.005401**	**0.001821**	**0**	0.085814	0.987653	**0.000795**	**0**
Day 6	**FY23**	**0**	**0.016302**	**0.019005**	0.321062	**0**	0.053052	0.089544	**0.024726**	**0**	0.077704	0.056762	0.106669
	FLO1	**0.016302**	**0**	**0.004551**	**0.017752**	0.053052	**0**	**0.014796**	0.150582	0.077704	**0**	**0.029639**	**0.033215**
	FLO5	**0.019005**	**0.004551**	**0**	**0.025518**	0.089544	**0.014796**	**0**	**0.047154**	0.056762	**0.029639**	**0**	**0.017132**
	FLO11	0.321062	**0.017752**	**0.025518**	**0**	**0.024726**	0.150582	**0.047154**	**0**	0.106669	**0.033215**	**0.017132**	**0**
Day 10	**FY23**	**0**	**0.013631**	**0.002186**	**0.007645**	**0**	**0.020252**	0.356845	**0.018882**	**0**	**0.043138**	**0.009584**	**0.003509**
	FLO1	**0.013631**	**0**	**0.005514**	**0.011481**	**0.020252**	**0**	**0.033059**	**0.021416**	**0.043138**	**0**	**0.009535**	**0.025863**
	FLO5	**0.002186**	**0.005514**	**0**	**0.01417**	0.356845	**0.033059**	**0**	**0.005229**	**0.009584**	**0.009535**	**0**	**0.047311**
	FLO11	**0.007645**	**0.011481**	**0.01417**	**0**	**0.018882**	**0.021416**	**0.005229**	**0**	**0.003509**	**0.025863**	**0.047311**	**0**
