# Supplementary material for: Modifying Saccharomyces cerevisiae Adhesion Properties Regulates Yeast Ecosystem Dynamics
Source: mSphere. 2018 Oct 24;3(5):e00383-18. doi: 10.1128/mSphere.00383-18 (PMC6200983; doi:10.1128/mSphere.00383-18)
Supplement: Supplementary file 5 [file sph005182668st5.docx]

		L. thermotolerans				*W. anomalus*				*S. cerevisiae*				*H. opuntiae*			
		**FY23**	FLO1	FLO5	FLO11	**FY23**	FLO1	FLO5	FLO11	**FY23**	FLO1	FLO5	FLO11	**FY23**	FLO1	FLO5	FLO11
DAY 2	**FY23**	**0**	0.130217	**0.00356**	0.43611	**0**	**0.018858**	0.058201	0.339031	**0**	**0.004129**	0.466257	**0.008983**	**0**	0.06204285	0.373741	0.062179
	FLO1	0.130217	**0**	**0.01124**	0.253103	**0.018858**	**0**	0.764134	**0.022239**	**0.004129**	**0**	**0.004574**	**0.001314**	0.062043	**0**	**0.048236**	0.438331
	FLO5	**0.00356**	**0.01124**	**0**	**0.018193**	0.058201	0.764134	**0**	**0.013765**	0.466257	**0.004574**	**0**	**0.012787**	0.373741	**0.04823556**	**0**	0.057941
	FLO11	0.43611	0.253103	**0.018193**	**0**	0.339031	**0.022239**	**0.013765**	**0**	**0.008983**	**0.001314**	**0.012787**	**0**	0.062179	0.4383312	0.057941	**0**
Day 6	**FY23**	**0**	**0.002261**	**0.022912**	0.160703	**0**	**0.037606**	0.215293	0.447385	**0**	0.072933	**0.021031**	0.829084	**0**	**0.01952354**	**0.034071**	0.259225
	FLO1	**0.002261**	**0**	**0.008268**	0.52503	**0.037606**	**0**	**0.003**	0.099039	0.072933	**0**	**0.021905**	0.133829	**0.019524**	**0**	**7.98E-05**	**0.037732**
	FLO5	**0.022912**	**0.008268**	**0**	0.268021	0.215293	**0.003**	**0**	0.870737	**0.021031**	**0.021905**	**0**	**0.000223**	**0.034071**	**7.9757E-05**	**0**	0.096483
	FLO11	0.160703	0.52503	0.268021	**0**	0.447385	0.099039	0.870737	**0**	0.829084	0.133829	**0.000223**	**0**	0.259225	**0.03773177**	0.096483	**0**
Day 10	**FY23**	**0**	0.257133	0.056463	**0.017476**	**0**	**0.011779**	**0.047743**	**0.001538**	**0**	**0.002095**	**0.043705**	0.081964	**0**	**0.03220946**	na	na
	FLO1	0.257133	**0**	**0.045178**	**0.032563**	**0.011779**	**0**	**0.032882**	**0.009617**	**0.002095**	**0**	**0.003059**	**0.004022**	**0.032209**	**0**	**0.032209**	**0.032209**
	FLO5	0.056463	**0.045178**	**0**	**0.030547**	**0.047743**	**0.032882**	**0**	**0.021102**	**0.043705**	**0.003059**	**0**	**0.022748**	na	**0.03220946**	**0**	na
	FLO11	**0.017476**	**0.032563**	**0.030547**	**0**	**0.001538**	**0.009617**	**0.021102**	**0**	0.081964	**0.004022**	**0.022748**	**0**	na	**0.03220946**	na	**0**
Day 16	**FY23**	**0**	**0.022137**	**0.003968**	0.091327	**0**	**0.016751**	**0.000163**	**6.65E-05**	**0**	**0.020825**	**0.049385**	**0.040801**	**0**	na	na	na
	FLO1	**0.022137**	**0**	**0.014457**	**0.041173**	**0.016751**	**0**	0.055809	**0.007729**	**0.020825**	**0**	**0.041173**	**0.008525**	na	**0**	na	na
	FLO5	**0.003968**	**0.014457**	**0**	**0.021165**	**0.000163**	0.055809	**0**	**2.01E-05**	**0.049385**	**0.041173**	**0**	**0.025772**	na	na	**0**	na
	FLO11	0.091327	**0.041173**	**0.021165**	**0**	**6.65E-05**	**0.007729**	**2.01E-05**	**0**	**0.040801**	**0.008525**	**0.025772**	**0**	na	na	na	**0**
